# Detection of Burst Suppression Patterns in EEG Using Recurrence Rate

**DOI:** 10.1155/2014/295070

**Published:** 2014-04-17

**Authors:** Zhenhu Liang, Yinghua Wang, Yongshao Ren, Duan Li, Logan Voss, Jamie Sleigh, Xiaoli Li

**Affiliations:** ^1^Institute of Electrical Engineering, Yanshan University, Qinhuangdao 066004, China; ^2^State Key Laboratory of Cognitive Neuroscience and Learning and IDG/McGovern Institute for Brain Research, Beijing Normal University, Beijing 100875, China; ^3^Center for Collaboration and Innovation in Brain and Learning Sciences, Beijing Normal University, Beijing 100875, China; ^4^Institute of Information and Science Engineering, Yanshan University, Qinhuangdao 066004, China; ^5^Department of Anesthesia, Waikato Hospital, Hamilton, New Zealand

## Abstract

Burst suppression is a unique electroencephalogram (EEG) pattern commonly seen in cases of severely reduced brain activity such as overdose of general anesthesia. It is important to detect burst suppression reliably during the administration of anesthetic or sedative agents, especially for cerebral-protective treatments in various neurosurgical diseases. This study investigates recurrent plot (RP) analysis for the detection of the burst suppression pattern (BSP) in EEG. The RP analysis is applied to EEG data containing BSPs collected from 14 patients. Firstly we obtain the best selection of parameters for RP analysis. Then, the recurrence rate (RR), determinism (DET), and entropy (ENTR) are calculated. Then RR was selected as the best BSP index one-way analysis of variance (ANOVA) and multiple comparison tests. Finally, the performance of RR analysis is compared with spectral analysis, bispectral analysis, approximate entropy, and the nonlinear energy operator (NLEO). ANOVA and multiple comparison tests showed that the RR could detect BSP and that it was superior to other measures with the highest sensitivity of suppression detection (96.49%, *P* = 0.03). Tracking BSP patterns is essential for clinical monitoring in critically ill and anesthetized patients. The purposed RR may provide an effective burst suppression detector for developing new patient monitoring systems.

## 1. Introduction

The electroencephalographic burst suppression pattern (BSP) consists of high amplitude bursts interrupted by low amplitude suppressions. It can be observed in different clinical conditions (head trauma, stroke, coma, anoxia, and hypothermia) [1, 2] and can also be induced by pharmacological agents such as anesthetics, analgesics, or antiepileptic drugs [3]. The BSP is a representative of the interaction between neuronal dynamics and brain metabolism. Each series of successive bursts can be viewed as an attempted recovery of basal cortical dynamics [4]. So, the BSP can be seen as a defined “reference point” during administration of anesthetic or sedative agents and is considered a reliable indicator of adequate cerebral-protection for various neurosurgical diseases. It is commonly used as a monitor for the titration of sedatives in order to achieve a maximum reduction of cerebral metabolic rate [5].

Many researchers have investigated methods for BSP detection. Early methods were based on the spectral analysis, such as the spectral edge frequency and the median frequency [6, 7]. Although these methods can successfully obtain the frequency and spectral characteristics of the BSP [8], they ignore the intense nonlinearity of the BSP, resulting in low accuracy of detection. The bispectral method was designed to distinguish the BSP in the EEG series, but it is based on a two-dimensional function, which requires complicated computational processes. A recent method based on the information theory and nonlinear time series analysis (approximate entropy) has been also developed [9]. This method evaluates the signal regularity in the EEG series for detection of the BSP. Actually, both the burst signal and suppression signal in the EEG series are surprisingly regular, so the approximate entropy can detect BSP in normal EEG series signals; however it cannot differentiate between the burst and suppression patterns. The nonlinear energy operator (NLEO) is a simple nonlinear method for BSP detection, which measures the energy in a single-component signal [10–12]. However, it is very sensitive to the exaction threshold selection. Therefore, a robust approach for the reliable detection of BSP remains elusive.

Recurrence quantification analysis (RQA) [13] can measure the complexity of a short and non-stationary characteristic signal with noise [14, 15]. Furthermore, it can analyze both linear and nonlinear time series to quantify the activity of a system irrespective of the numbers or dynamical nature of the individual sources [16]. Up to now, the RQA has been broadly applied in the analysis of physiological data [17–20]. In this study, we investigated whether it could be applied to the EEG for detection of the BSP.

The paper is organized as follows. In Section 2 the subjects and recordings, signal preprocessing, RQA methods, and statistical analysis are introduced. Then we provide the results for parameter selection and a comparison of different RQA measures. After choosing the best RQA parameter using statistical analysis, we compared its performance with a few existing BSP detection methods. After that we show the application of RQA measure to a long-term EEG records. At last we discuss some properties of the proposed method.

## 2. Materials and Methods

### 2.1. Subjects and EEG Recordings

The data used in this study were obtained from a previously reported study on dreaming during general anesthesia [21, 22]. Clinical trials registration is ClinicalTrials.gov identifier NCT00446212; Australian Clinical Trials Registry number is ACTRN12606000279527. Ethical committee review and patient written informed consent were obtained. Two experienced experts selected 14 patients whose EEGs include obvious burst suppression patterns out of a group of patients (300) recruited for the sleep data collection. These patients were adults (aged 18–50 yrs) who were American Society of Anesthesiologists' physical status I–III, presenting for elective noncardiac surgery under relaxant general anesthesia with endotracheal intubation. Patients were randomized to two groups before the surgery. Anesthesia was induced with propofol, fentanyl 1-2 *μ*g/kg, and a muscle relaxant and then maintained with either propofol or desflurane; and the patient's lungs ventilated for normocapnia. The maintenance hypnotic (either propofol or desflurane) was then titrated to aim for a bispectral index (BIS, Aspect Medical Systems Inc.) of 40–55. The EEG signal was obtained using the Aspect XP monitor (BIS-XP, Aspect Medical Systems Inc., Norwood, MA) with the proprietary electrode strip on the standard recommended prefrontal montage (FP2-FT7). The raw signal was digitized at 100/sec 14-bit precision and stored by a laptop computer. Offline analysis was performed using the MATLAB software (version 7, MathWorks Inc.).

### 2.2. EEG Preprocessing

Artifacts in scalp EEG recordings mainly come from eye movement, muscle activity, and power frequency noise. To reduce these artifacts, the following steps are carried out. First, statistical mean and standard deviation methods were used to remove the outlier points. Then, a stationary wavelet transform [23] was utilized to reduce the electrooculogram (EOG) artifact by setting an appropriate threshold. Finally, the two-way least-square finite impulse response (FIR) at a zero-phase forward and reverse operation [24] was applied to reduce 0–0.5 Hz baseline drift.

### 2.3. BSP Detection

**Figure 1:**
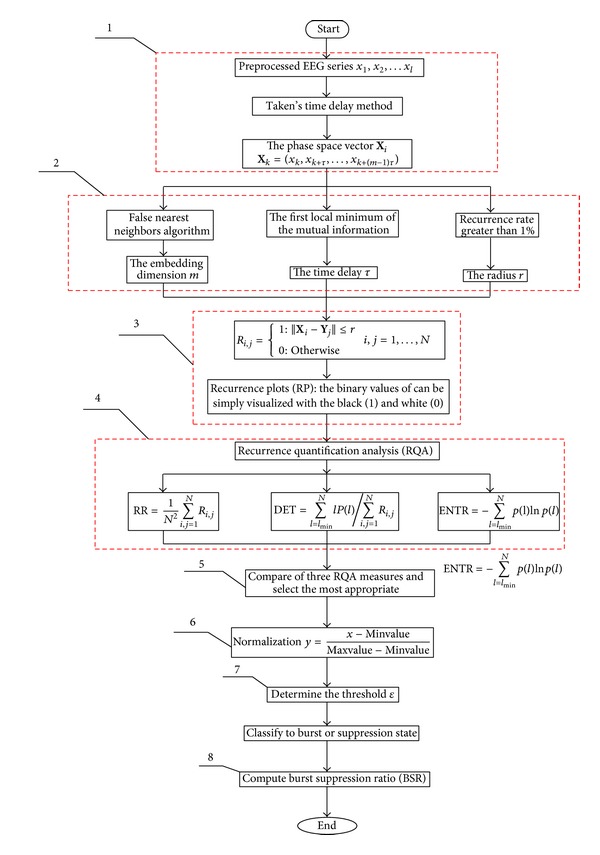
The block diagrams of EEG signal processing.

The process of BSP detection is depicted with a block diagram in Figure 1.

*Block 1.* For a given time series *x*_1_, *x*_2_,…, *x*_*l*_ construct the phase space vector **X**_*i*_ using a time delay method, **X**_*k*_ = (*x*_*k*_, *x*_*k*+*τ*_,…, *x*_*k*+(*m*−1)*τ*_) [25], based on the observations *x*_*k*_. Here *k* = 1,2,…, *N* − (*m* − 1), *τ* is the delay time, and *m* is the embedding dimension.

*Block 2*. Determine the parameters *m*, *τ*, and *r*.

**Figure 2:**
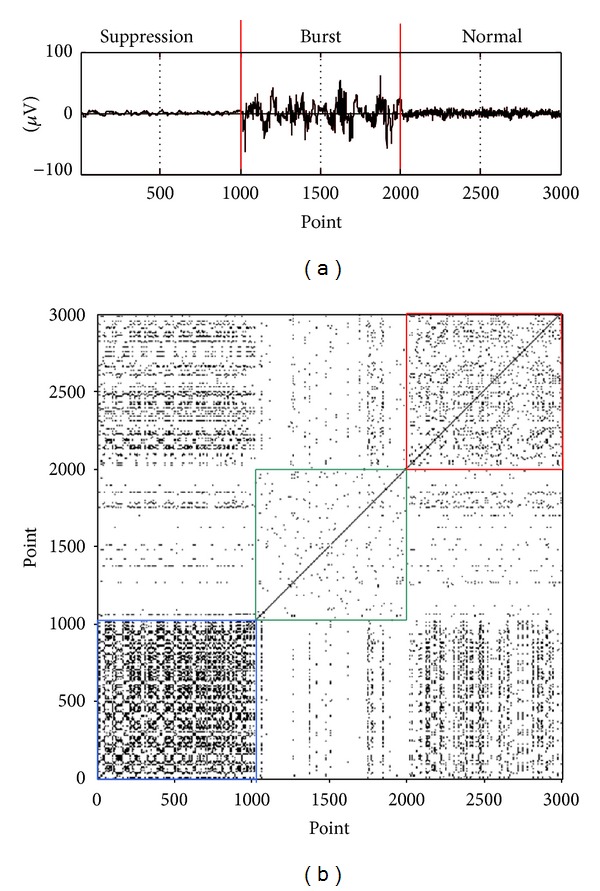
(a) A composite EEG signal from a patient. It consists of suppression (1000-point), burst (1000-point), and normal (1000-point) records artificially joined together; (b) different RP patterns during suppression, burst, and normal states, respectively. The blue box represents the suppression, the green box the burst, and the red box the normal state.

*Block 3*. Use recurrence plot (RP) to visualize the time dependent behavior of orbits **X**_*i*_ in a phase space. It is shown that RP can describe dynamical characteristics of burst suppression patterns. The key step of RP is to calculate the following *N* × *N* matrix:

(1) Ri,j={1:||Xi−Yj||≤r0:Otherwisei,j=1,…,N,

where *N* is the number of points in the times series for analysis, ||·|| is the norm (the *L*_*∞*_-norm is selected because it is computationally faster and allows the studying of some features in RPs analytically), and *r* is the cutoff distance defining an area centered at **X**_*i*_. As can be seen in Figure 2, the EEG signal is comprised of three segments, 1000-point suppression, 1000-point burst, and 1000-point normal EEGs from the first patient. RP forms are different for EEG segments during suppression, burst, and normal states. The blue box corresponding to the suppression shows thick black dots; the green box to the burst shows sparse dots; and the red box to the normal state shows uniform dots, which is similar to that of a white noise.

*Block 4*. Analyze the recurrence point density in RPs. The procedure is known as recurrence quantification analysis (RQA). More details of RP and RQA can be found in [26, 27]. First we introduce the simplest measure of RQA, RR, which is a measure of the density of recurrence points which simply counts the black dots in the RP. RR is calculated by

(2) $$\begin{array}{c} \text{RR}=\frac{1}{N^{2}}\overset{N}{\underset{i,j=1}{\sum }}R_{i,j}. \end{array}$$

The ratio of recurrence points on the diagonal structures to all recurrence points is called determinism (DET). The DET is a determinism (or predictability) measure of a system, calculated by

(3) $$\begin{array}{c} \text{DET}=\frac{\sum_{l=l_{\min}}^{N}lP\left(l\right)}{\sum_{i,j=1}^{N}R_{i,j}}, \end{array}$$

where *P*(*l*) is the frequency distribution of the lengths of the diagonal structures in the RP and *l*_min⁡_ is the threshold, which excludes the diagonal lines formed by the tangential motion of a phase space trajectory.

The ENTR is considered as a complexity measure of a deterministic structure in a dynamical system. The ENTR refers to the Shannon entropy of the frequency distribution of the diagonal line lengths. The more complex the deterministic structure, the larger the ENTR value. ENTR is calculated as

(4) $$\begin{array}{c} \text{ENTR}=-\overset{N}{\underset{l=l_{\min}}{\sum }}p\left(l\right)\ln p\left(l\right). \end{array}$$

*Block 5*. Compare the three RQA measures and select the most appropriate BSP index. The details are explained later in results.

*Block 6.* Normalize the index to eliminate individual differences. The normalized linear formula is calculated by

(5) $$\begin{array}{c} y=\frac{x-\text{mean}\left(x\right)}{\max\left(x\right)-\min\left(x\right)}. \end{array}$$

*Block 7*. Determine the threshold *ε* for detecting the BSP. The details are explained later in results.

*Block 8*. Calculate the burst suppression ratio (BSR). BSR represents the intensity of the burst suppression pattern in the long-term EEG recordings [28]. It is calculated as

(6) $$\begin{array}{c} \text{BSR}=\frac{\text{Total}\;\text{time}\;\text{of}\;\text{suppression}}{\text{epoch}\;\text{length}}\times 100\%. \end{array}$$

### 2.4. Statistical Analysis

To test the performance of the three RQA indexes RR, DET, and ENTR to detect BSP in the EEG series, the one-way ANOVA and multiple comparison tests were performed on averaged RR values. We also compared RR and NLEO for detecting and classifying BSP with confusion matrixes [29, 30], which contains information about actual and predicted classifications done by a classification system.

## 3. Results

### 3.1. Choice of the Parameters

**Figure 3:**
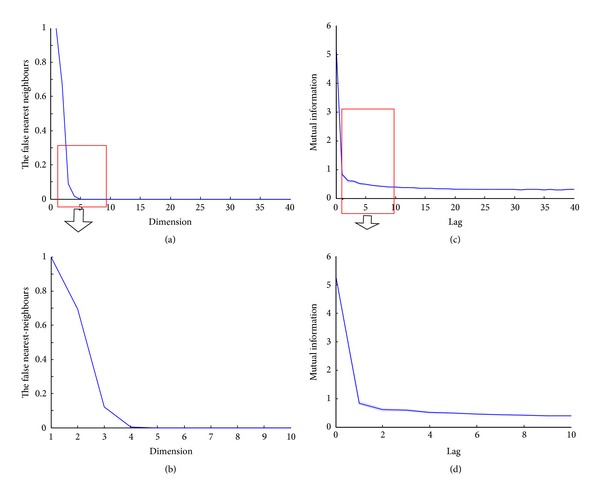
The embedding dimension and delay time of the EEG signals during the burst suppression state. (a) The false nearest neighbors versus the dimension with scales from 0 to 40. (b) The local plot of (a) with the dimension scales from 1 to 10. (c) The mutual information versus the delay time with scales from 0 to 40. (d) The local plot of (c) with the delay time scales from 1 to 10.

Prior to calculating a recurrent plot index from EEG data, the phase space reconstruction should first be determined. In consideration of the non-stationary characteristic of the EEG signals exhibiting rather sudden changes of state, the notion of a “correct” embedding or delay is inappropriate—as demonstrated by Grassberger and Schreiber [29]. Therefore, we determined the embedding dimension of EEG signals by using the false nearest neighbors algorithm [31].  Figure 3(a) shows the false nearest neighbors versus the dimension from 0 to 40, and the dimension is approximately 5 at the first local minimum of false nearest neighbors.  Figure 3(b) shows a focused view of Figure 3(a). It shows that the false nearest neighbors become stable for *m* ≥ 4; thus *m* = 4 would be the optimal choice of embedding dimension for the phase space reconstruction.

The first local minimum of the mutual information measure was used to determine the time delay parameter [32, 33]. This method provides a reconstruction which maximally “unfolds” the dynamics.  Figure 3(c) shows the mutual information versus the lag time 0 to 40, and the delay time *τ* is approximately 5 at the first local minimum of mutual information. From the focused view in Figure 3(d) we see that *τ* = 3 is a preferred value for the phase space reconstruction.

**Figure 4:**
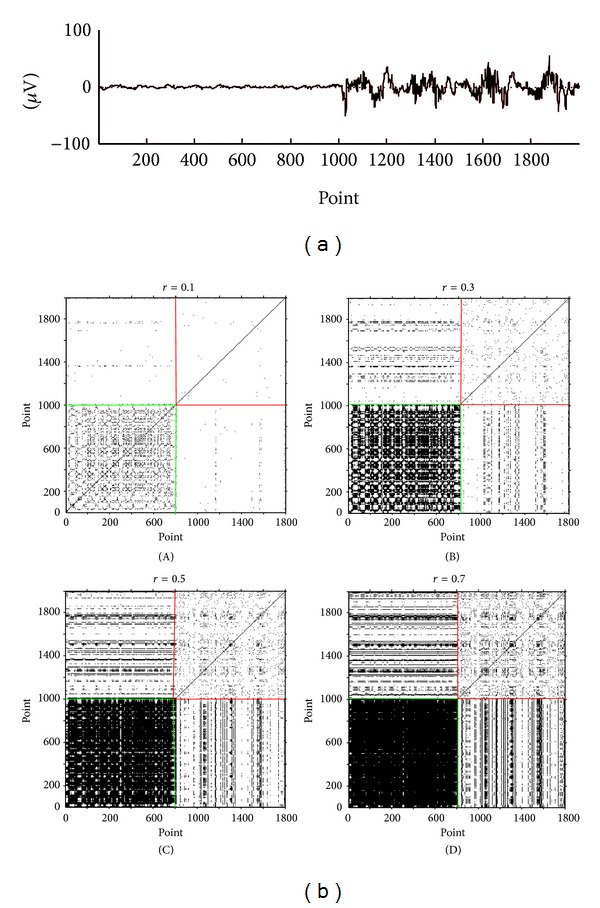
(a) An EEG signal consists of suppression and burst; (b) the different RP under four different radiuses for the signals in (a). (A) *r* = 0.1, (B) *r* = 0.3, (C) *r* = 0.5, and (D) *r* = 0.7 with a dimension of 4 and a delay of 3.

Another crucial parameter of RP is the radius *r*. Conventionally *r* is adjusted so that there would be sufficient numbers of recurrence values to make the recurrence rate larger than 1%.  Figure 4 shows the different RP under different radius for an identical typical time series composed of 10 s suppression and 10 s burst. When *r* = 0.1, the RP for the suppression part in the green box does not show an obvious recurrent state. But when *r* = 0.5 and *r* = 0.7, the green box of RP for the suppression part is filled with black dots and red box for the burst part in the red box shows some undesirable regular black dots. Only when *r* = 0.3 does the RP for suppression part show the required recurrent state. So *r* = 0.3 is an appropriate choice of the radius.

### 3.2. Comparison of Three RQA Measures

**Figure 5:**
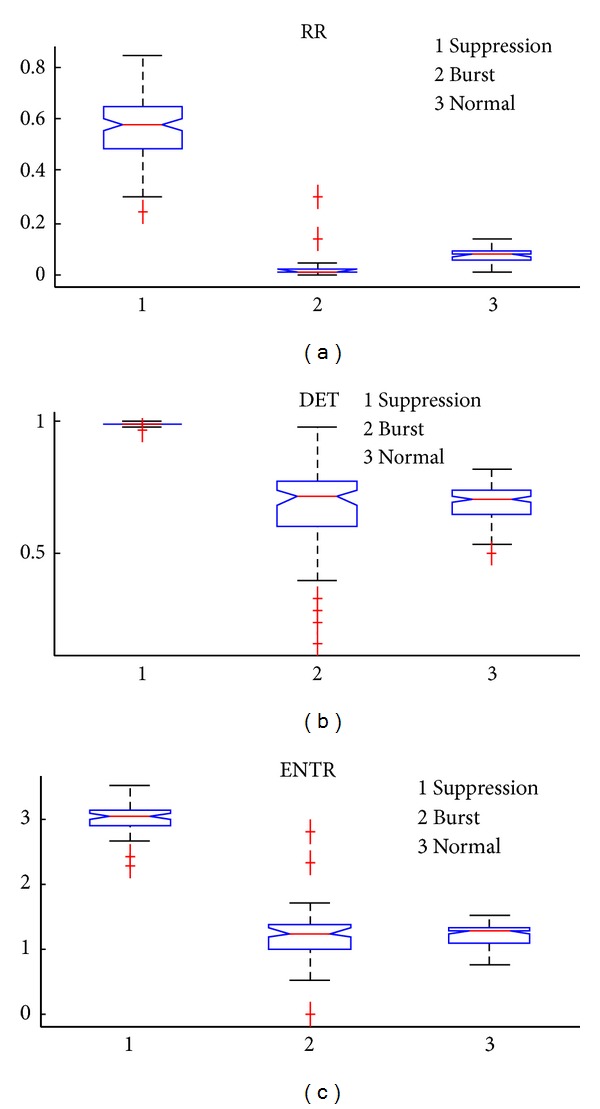
The boxplot of three different indexes at the burst suppression normal states. (a) The RR index, (b) The DET index. (c) The ENTR index.

The comparison of the three RQA measures, and, hence, selection of the BSP index, is another important issue.  Figure 5 shows the statistics of corresponding RR, DET, and ENTR calculated on an EEG series composed of 1000-point suppression, 1000-point burst, and 1000-point normal signals (see Figure 1).

As can be seen in Figure 5, all the three measures could readily distinguish the burst and suppression patterns. However, DET and ENTR do not show significant differences between the suppression and normal states.

To evaluate the performance of the three RQA indexes, we applied the one-way repeated measure ANOVA and multiple comparisons. As shown in Table 1, the difference between values at different states for all indices is all significant (*P* < 0.001). However, the RR has a larger *F* value and thus is the best at the *P* < 0.001 level of probability.

Multiple comparison tests showed that all three indices could distinguish between the burst and suppression states and the burst and normal states (difference of mean >0). However only the RR measure could distinguish between the suppression and normal states (the other two indexes' difference contains 0 and thus are not significant). So the RR measure was chosen to be the index of the BSP identification.

### 3.3. Selection of the Threshold *ε*

In order to detect BSP quantitatively and automatically, the optimal value of threshold *ε* needs to be determined. Differences in EEG amplitude between individual recordings will influence the threshold selection and reduce the versatility of the algorithm. So first we applied the normalized linear formula to the RR index to reduce individual differences. Then all the signals were analyzed statistically to look for the appropriate threshold. In the suppression state, the RR shows the highest values (0.185 to nearly 1). In the burst state the RR shows the lowest values (<0.055), whilst in the normal state the values are intermediate (0.055 to 0.185). Based on these values, the suppression threshold is set to 0.185 and the burst threshold to 0.055. Considering the influence of noise, four successive RR values exceeding the above thresholds are required before a burst or suppression is recognized.

### 3.4. Comparison of BSP Detection Methods

Four methods (spectral analysis, bispectral analysis, approximate entropy, and NLEO) have been employed for the detection of BSPs. In the following section, the RR is compared with the above mentioned four methods.

**Figure 6:**
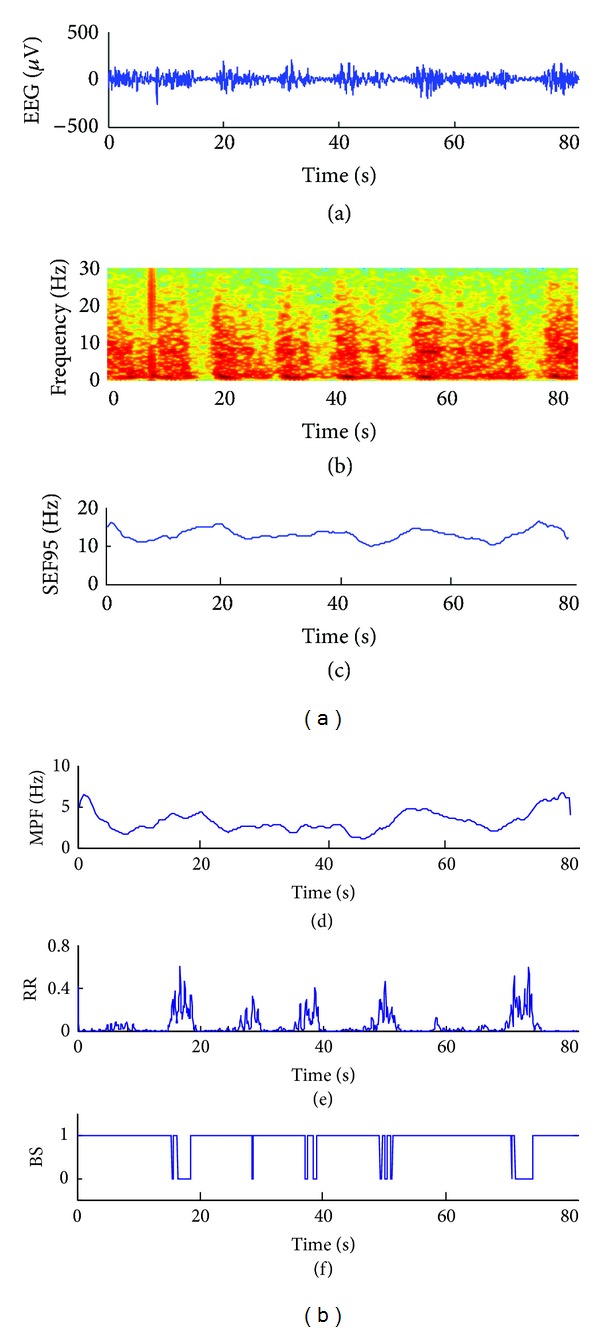
Comparison between the RR method and the spectral analysis based methods for the BSPs detection. (a) A burst suppression interval EEG signal of 80s, (b) frequency spectrum, (c) spectral edge frequency 95 parameter, (d) median electroencephalogram frequency parameter, (e) the RR index, and (f) the BS index of RR.

First, we discuss the spectral analysis based methods.  Figure 6(a) shows an example of 80 s burst suppression EEG signal. Even though the frequency spectrum of the EEG signal characterizes the suppression with vertical green regions in Figure 6(b), the spectral edge frequency 95 and median electroencephalogram frequency do not change reliably with the different patterns and therefore fail to detect the burst suppression pattern (Figures 6(c)-6(d)). Conversely, the RR and the detected bursts are clearly shown in Figures 6(e)-6(f).

**Figure 7:**
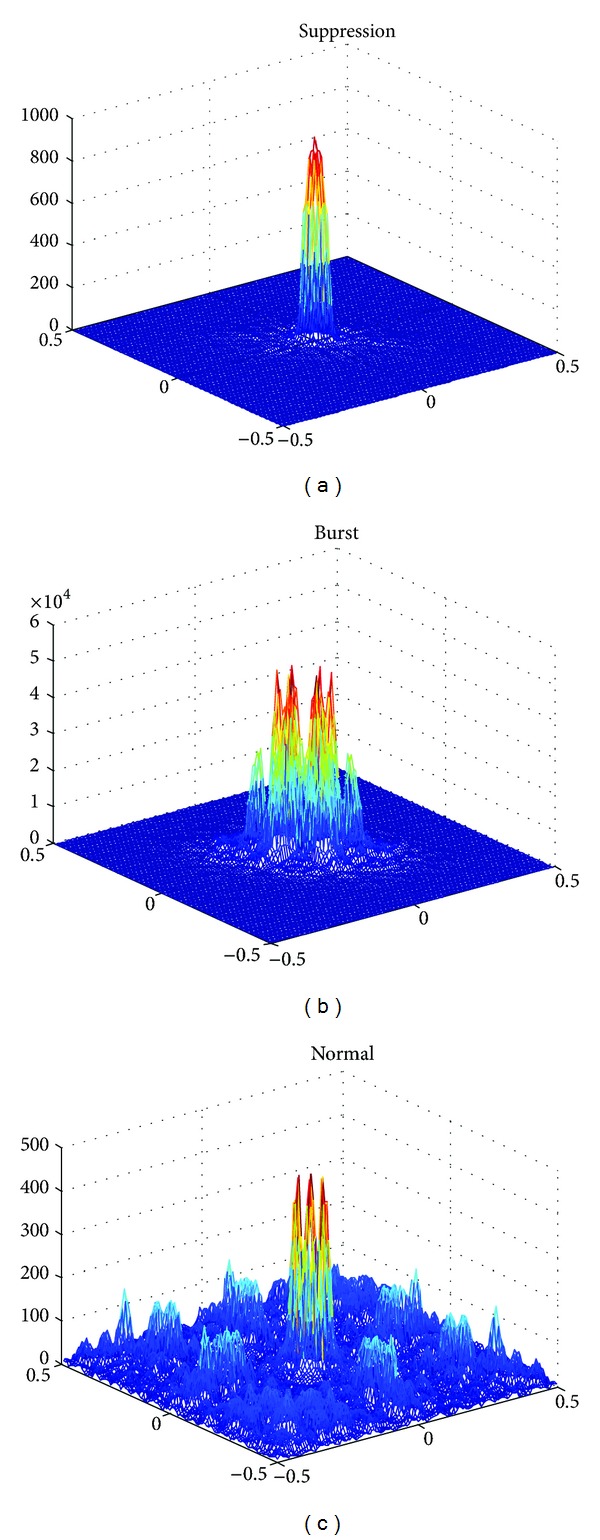
The bispectrum of burst, suppression, and normal states, respectively.

The bispectral analysis is another method used for BSP detection. In Figure 7, the bispectrum at the suppression, burst, and normal states is plotted in the frequency-frequency domain, where some differences in phase coupling between some frequency bands can be seen corresponding to the different EEG patterns. However it is noted that the bispectral analysis is a complicated method and is very sensitive to noise, which limits its usefulness.

**Figure 8:**
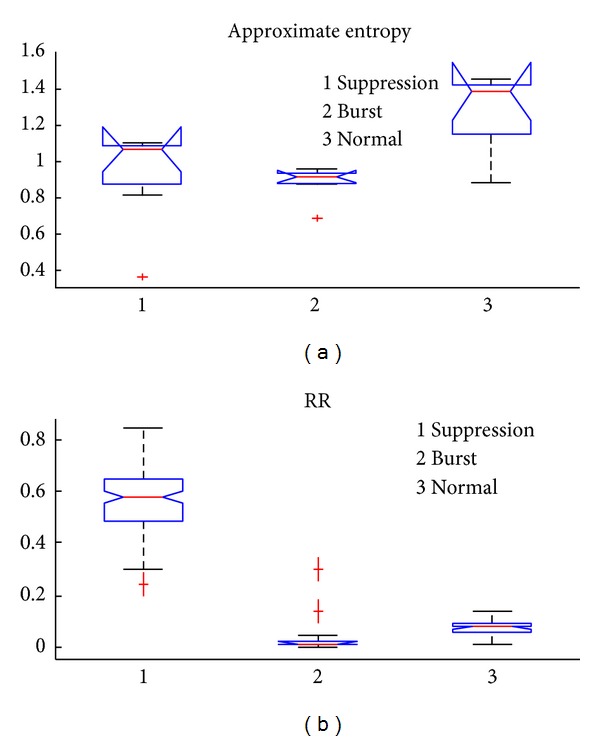
The boxplot of two different indexes at the burst, suppression, and normal states. (a) Approximate entropy. (b) The RR index.

Approximate entropy has also been proposed to detect the BSP [9]. As shown in Figure 8, the normal state boxplot is generally different from the burst and suppression states, while the boxplots of suppression and burst states are overlapping. Thus approximate entropy can distinguish between the normal state and burst/suppression but could not reliably discriminate between the burst and suppression states. Conversely, the RR index boxplot shows no overlap between the burst, suppression, and normal states. Thus the RR index is superior to approximate entropy for burst and suppression detection.

Nonlinear energy operator (NLEO) is one of the most popular methods in burst suppression pattern detection. The NLEO and RR methods were used to analyze the EEG signals of all subjects and the statistical results are shown in Table 2. The confusion matrix between manual classification versus RR and NLEO, respectively, is listed in Table 3. The RR method for suppression detection has a higher agreement with manual results than that of the NLEO (*P* = 0.03, Fisher's exact test).

### 3.5. Application to EEG

To assess the changes of RR over time, the RR of the long-term EEG records for 14 subjects was obtained. The RR was calculated on 10 s EEG moving window, and parameters for RR were *m* = 4, *τ* = 3, and *r* = 0.3.

**Figure 9:**
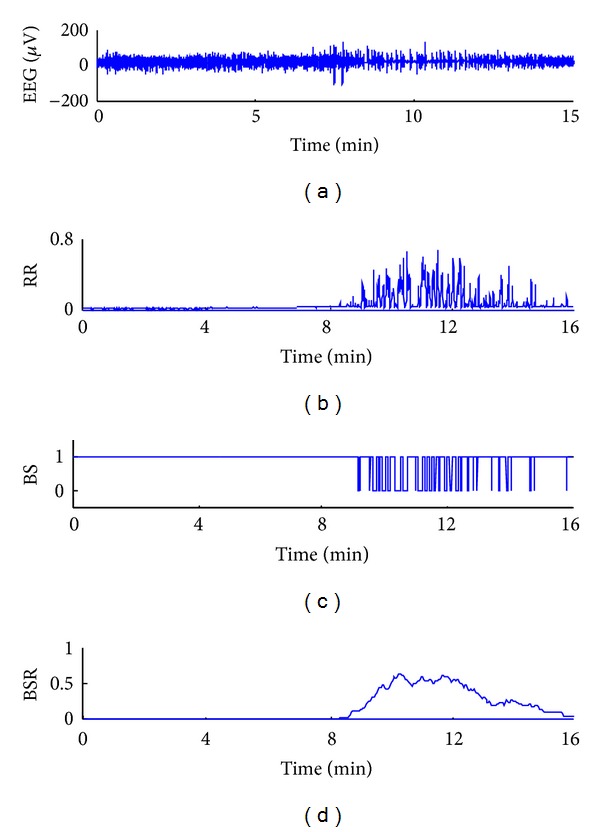
(a) The long-term EEG recordings with burst suppression patterns. (b) The observation of RR over the entire EEG recordings. (c) Suppression is represented with 0 and burst with 1 to obviously distinguish the two states. (d) The BSR is calculated.

Figure 9(a) shows a 16 min EEG recording with burst suppression patterns from one subject. RR tracks the changes of the EEG activities over time (Figure 9(b)). The RR values are high during the suppression phase. During the burst phase, the RR decreases to a small value. The classified burst/suppression states are plotted in Figure 9(c), where the suppression is represented with 0 and the burst with 1. The time course of BSR is shown in Figure 9(d).The BSR reveals a progressive increase and then a decrease from 8 min to 16 min. This correlated well with the underlining patient procedure.

## 4. Discussion

Previously, several methods have been proposed to detect the burst suppression pattern of EEG signals [34, 35]. However, we still need to overcome several drawbacks in the existing methods for constructing a practical burst suppression detection system in EEG recordings. In this study, recurrent plot (RP) is proposed to distinguish burst and suppression states in EEG. To our knowledge, this is the first work to use the RP to detect the burst suppression patterns in EEG. RR increased at suppression state in our study. This result is consistent with Ching et al.'s result. They proposed an important principle that complexity decreases and recurrence increases during suppression compared to the burst state [4].

The advantage of RR is that it does not have constraints and assumptions, because it only counts similar events in an embedded space [36]. Therefore, RR can be used to analyze a wide range of linear or nonlinear, stationary or non-stationary characteristic, and noisy or noiseless time signals [16]. The “traditional” methods (such as spectral analysis) fail to detect the different states within an EEG record, as shown in Figure 6. Other nonlinear methods need a long, stationary, and noiseless EEG time series and thus are not very suited to detect transient characteristics in the EEG series, for example, burst and suppression patterns. The entropy method is used to evaluate a signal's regularity, whereas the burst and suppression EEG signals are both relatively regular. This explains why approximate entropy is unable to differentiate between the burst and suppression patterns.

The RR index is not very sensitive to choice of threshold, because it is based on the distance of different dots and is independent of the signal amplitude. Amplitude differences between individual recordings were eliminated through the normalization of the RR index. In contrast, the NLEO method is very sensitive to the choice of an appropriate threshold. Thus we would conclude that the RR method is more robust than other methods and is suitable for further development of a BSP detector.

## Figures and Tables

**Table 1:** One-way ANOVA and multiple comparison test of three RQA indexes.

	One-way ANOVA		Multiple comparison test		
	*F*	*P*	[suppression burst]	[suppression normal]	[burst normal]
RR	**1282.82**	<0.001	[0.51 0.56]	[0.46 0.51]	[−**0.08** −**0.02**]
DET	246.10	<0.001	[0.27 0.35]	[0.25 0.32]	[−0.06 0.01]
ENTR	920.06	<0.001	[1.75 1.98]	[1.68 1.91]	[−0.18 0.04]

**Table 2:** The classification of manual, NLEO, and RR methods.

	Manual	NLEO	RR
Burst	427	459	426
True burst	—	408	412
False burst	—	51	14

Suppression	427	395	428
True suppression	—	376	413
False suppression	—	19	15

**Table 3:** The confusion matrix between the NLEO and the RR.

	NLEO		RR	
	Burst (%)	Suppression (%)	Burst (%)	Suppression (%)
Manual				
Burst (%)	95.50	4.50	**96.49**	3.51
Suppression (%)	11.94	88.06	3.28	**96.72**
